# Python algorithms in particle tracking microrheology

**DOI:** 10.1186/1752-153X-6-144

**Published:** 2012-11-27

**Authors:** Timo Maier, Tamás Haraszti

**Affiliations:** 1 Max-Planck Institute for Intelligent Systems, Advanced Materials and Biosystems, Heisenberg str. 3, 70569 Stuttgart, Germany; 2 Biophysical Chemistry, Institute of Physical Chemistry, University of Heidelberg, Im Neuenheimer Feld 253, 69120 Heidelberg, Germany

**Keywords:** Particle tracking microrheology, Numerical conversion method, Software library, Dynamic interpolation

## Abstract

**Background:**

Particle tracking passive microrheology relates recorded trajectories of microbeads, embedded in soft samples, to the local mechanical properties of the sample. The method requires intensive numerical data processing and tools allowing control of the calculation errors.

**Results:**

We report the development of a software package collecting functions and scripts written in Python for automated and manual data processing, to extract viscoelastic information about the sample using recorded particle trajectories. The resulting program package analyzes the fundamental diffusion characteristics of particle trajectories and calculates the frequency dependent complex shear modulus using methods published in the literature. In order to increase conversion accuracy, segmentwise, double step, range-adaptive fitting and dynamic sampling algorithms are introduced to interpolate the data in a splinelike manner.

**Conclusions:**

The presented set of algorithms allows for flexible data processing for particle tracking microrheology. The package presents improved algorithms for mean square displacement estimation, controlling effects of frame loss during recording, and a novel numerical conversion method using segmentwise interpolation, decreasing the conversion error from about 100% to the order of 1%.

## Background

Particle tracking microrheology is a modern tool to investigate the viscoelastic properties of soft matter, for example, biopolymers and the interior, or the membrane of living cells [1,2] on the microscopic scale. Though embedding tracer particles into such a sample alters the local structure, this method is still considered non-invasive and provides important information not available by other methods [1-4].

The physical background of the method lies in the thermal motion of the tracer particle, which can be connected to the viscoelastic properties of the local environment through the generalized Langevin equation [5,6]. Neglecting the inertia term, which contributes to frequencies in the megahertz range, and assuming that the memory function is linearly related to the frequency dependent viscosity of the medium (through a generalized Stokes-Einstein relation) [3,5-8], the mean square displacement (MSD) of the particle can be directly related to the creep compliance as:

(1) $$J(\tau )=\frac{3\Pi a}{N_{D}k_{B}T}<\Delta r^{2}(\tau )>,$$

where *τ* denotes the time step in which the particle moves Δ*r* distance, <Δ*r*^2^(*τ*)> the mean square displacement, *N*_*D*_ the dimensionality of the motion (usually *N*_*D *_= 2 for particle tracking digital microscopy), *k*_*B *_the Boltzmann constant, *T* is the absolute temperature and *a* the particle radius, respectively.

Active microrheology (using optical or magnetic tweezers) and macroscopic rheometry commonly characterize the sample elasticity with the frequency-dependent complex shear modulus, *G*^∗^(*ω*), which is a complex quantity [4,9,10]. Its real part is known as the storage modulus *G*^*′*^(*ω*) and the imaginary part is the loss modulus, *G*^*′′*^(*ω*). While *J*(*t*) is a description in the time domain, *G*^∗^(*ω*) is an equivalent characterization in the frequency domain. The two types of description are equivalent and interconnected with the relation:

(2) $$G^{*}(\omega )=\frac{1}{i\omega \tilde{J}(\omega )},$$

where $\tilde{J}(\omega )$ is the Fourier transform of *J*(*t*). Assuming that the particle tracks are previously obtained, the frequency dependent complex shear modulus *G*^∗^(*ω*) can be derived using equations (1) and (2) after calculating the mean square displacement.

There are two major algorithm libraries available on the Interned addressing data handling for microrheology: the algorithm collection of J. Crocker et al. written in the interactive data language (IDL) [11], which was translated to Matlab and expanded by the Kilfoil lab [12]. A separate stand alone algorithm is provided by M. Tassieri for calculating the complex shear modulus from the creep compliance, written in LabView [13]. However, an extensible integrated framework relying on freely accessible software and source code integrating multiple conversion methods is not yet available.

In this paper we present a software package written in the interpreted programing language Python (http://www.python.org) collecting functions to support particle tracking microrheology related calculations, with emphasis on those parts providing enhanced functionality. This library is meant to be an open source, platform independent, freely extendable set of algorithms allowing to extract rheology information from particle tracks obtained previously.

**Figure 1:**
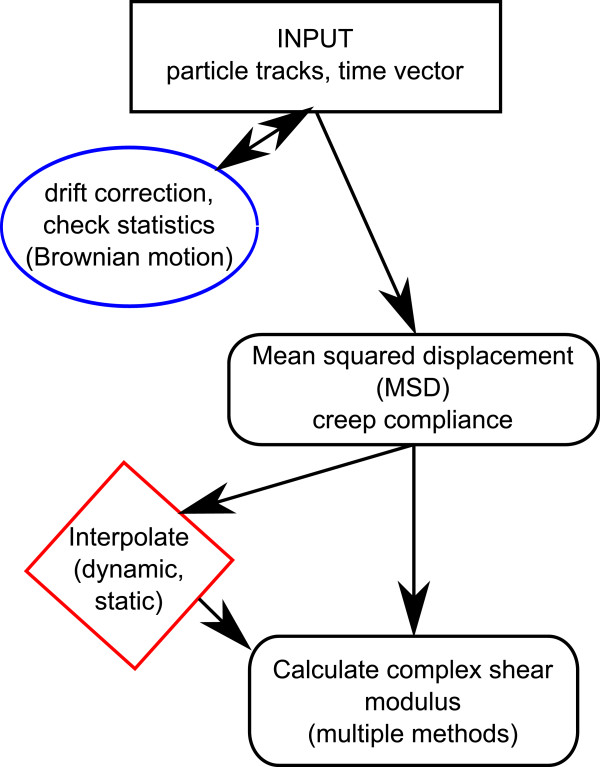
**Process flow chart of microrheology data.** The fundamental processing steps in particle tracking microrheology as followed by the *ProcessRheology.py* script. There are several parameters that may affect the details of the process, including the sampling in the MSD calculation and which way the complex shear modulus is calculated (see the main text for details). In this article we focus mainly on the later tree steps in the process: the MSD calculation and conversion methods.

The Rheology software library contains two example scripts. One called *ProcessRheology.py*, a configuration driven program performing all processing steps from particle trajectory inputs, thus presenting the various capabilities of the library (Figure 1). This program can be employed as a self-standing calculator or its code can be used as a template for the user testing the Rheology library. The other is the *Function-test.py*, containing test calculations, and which was also employed to produce the figures in this article (see the content of the Additional file 1).

## Implementation

### Dependencies

The software depends on the following Python packages for calculations and displaying results: 

• **Numpy:** a library for array manipulation and calculations [14];

• **Scipy:** the Python scientific library, from which we used the gamma function and the nonlinear least squares fitting function [14];

• **Matplotlib:** a Matlab-like plotting library to generate information graphs of the results [15].

After installation, its functions are available using following import command within the interpreter or a Python script:

from Rheology import ⋆

### Data presentation, data format

Instead of defining individual classes for each data type (MSD, creep compliance and complex shear modulus), an alternative technique in Python is to employ generalized lists, the so called dictionaries or ‘dicts’. A dict is a container class holding data which are indexed with arbitrary keys (marked by quotes in this paper). This is a very general and flexible data structure, which we used for all data in the Rheology package. For example, MSD dicts contain keys “tau” and “MSD” referring to arrays holding the time lag and the mean square displacement data, respectively.

### Particle trajectories

Particle tracking microrheology starts with the imaging experiments and the image treatment in a strict sense, however, obtaining particle trajectories from video microscopy is well described in the literature [16-20] including the statistical difficulties of the process [21-23]. There are various implementations of particle tracking algorithms available in IDL (see the same website as for the rheology code) [11,24], Matlab [12,25], LabView [20,26] or C++ languages [27]. An implementation of the Grier method [16] is also translated to Python [28].

Thus, we start our discussion by extracting rheological information from the particle trajectories, leaving the implementation of data input/output to the user. As a good starting template, we recommend the *ReadTable()* and *SaveTable()* functions in the *ProcessRheology.py* example script.

### Drift correction

Several experimental systems show a drift: a slow oriented motion of the sample versus the imaging frame, which is caused by various factors of the experimenting apparatus. To remove this drift, which is independent of the sample, there are multiple possibilities one may consider. If a reference bead bound to the sample is available, its trajectory provides the drift itself. If multiple particles are tracked in the same time interval, an average trajectory may be calculated and used as a reference. If these possibilities are not available, one has to consider whether the long time motion is due to drift or it is a characteristic of the investigated sample, because subtracting it changes the resulted long time lag (low frequency) part of the viscoelastic characteristics.

The simplest way of data treatment in such cases is to calculate a smoothened trajectory: e. g. using a running average, and either subtract this smoothened data or, in one further step, fit a low order polynomial to the smoothened data and subtract the fitted values from the trajectory.

A very simple implementation is available for two dimensional data sets as:

GetData(timestamps, poslist, indx=0,

order=3,resolution=0.1434, Nd= 1000)

This algorithm takes a position list (parameter *poslist*), which is a list of dicts, each describing the positions of a particle. The *indx* parameter is used to select one of them. A position dict contains “X”, and “Y”, which are arrays of the *x* and *y* positions. The dict also contains an index array denoted with key “indx”, identifying the image index (serial number) of the given positions. Using this index allows the tracking algorithm to miss individual frames (e. g. when the particle drifted out of focus). This index is also used to define the time point of a position, either by identifying the corresponding time stamps, provided in seconds in the *timestamps* array, or if this variable is set to None, multiplying the index by the optional *tscale* parameter. The *Nd* parameter gives the number of points used in the running average and the *order* parameter identifies the order of the polynomial to be fitted for drift correction. If *Nd* is set to −1, the running averaging is off, and if *order* is -1, the drift correction is turned off. The *resolution* is used to scale the coordinates to micrometers (the same value for both coordinates).

#### Mean square displacement (MSD)

Characterizing a soft sample using particle tracking microrheology strongly relies on the determination of the mean square displacement. Calculating the MSD has two possible sets of assumptions: 1.) ensemble averages are based on recording many tracer particles and assuming homogeneity across the sample. This is the averaging method considered in theories, and has the advantage allowing estimation of the time-dependent aging of the system. Technically this can be achieved by using video recording-based particle tracking, where the number of tracers can be increased to the order of tens. 2.) Assuming ergodicity, one can switch from ensemble averaging to time averaging. This is very important for systems which are not homogeneous and for cases where only few particles (1−5) can be observed at a time. For samples of biological origin, time averaging is more suitable because these samples are seldom homogeneous.

**Figure 2:**
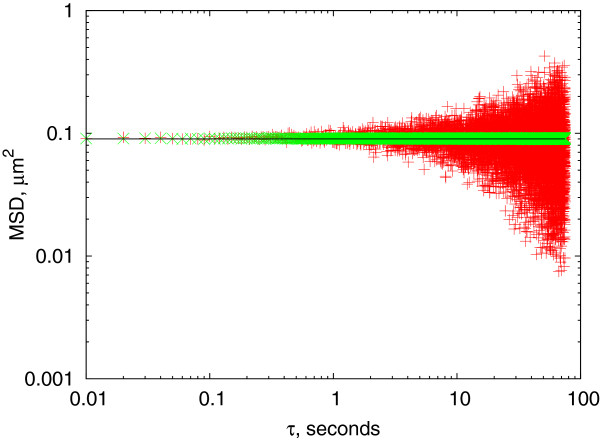
**mean square displacement data calculated from simulated data.** The distribution of X and Y positions were generated based on a normal distribution with *σ *= 0.15*μm*. The theoretical MSD is constant at 0.09*μm*^2^(black line). Data with non-overlapping intervals (red + symbols) show higher scattering, the ones calculated with overlapping intervals (green ×) show a much lower error.

Calculating the time average is done by splitting up the trajectory into non-overlapping parts and averaging their displacement. Because the number of intervals decreases with an increasing lag time, this method has very high error for large lag time values. Alternatively, it is also possible to split the trajectory into overlapping regions and then do the averaging. The resulting statistical errors follow a non-normal distribution, but it has been shown that using overlapping segments the resulted MSD may show improved accuracy [29,30] when using lag time values up to about the quarter of the measurement length (*N*/4 for *N* data points). The results of a test calculation using positions randomly calculated from a normal distribution with standard deviation of *σ*_*X *_=* σ*_*Y *_= 0.15*μm *in both X and Y directions are presented in Figure 2. The MSD oscillates around the theoretical value of $<\Delta r{\left(\tau \right)}^{2}>=2(\sigma_{X}^{2}+\sigma_{Y}^{2})=0.09\mu m^{2}$ as expected, but the data calculated using overlapping intervals show visibly better accuracy.

msd = MSD(positionlist, tau= 0)

*MSD*() is the function to calculate the mean square displacement from a single trajectory. The data points are presented as a two dimensional array *positionlist*, containing coordinates ((*xy*) or (*xyz*)) in each row.

The second parameter (*tau*) is used to generate the lag time values (steps) for which the MSD is calculated. This parameter can take various values: If an array or list of integer values are given, then those are used as index step values. If a single integer is given between 0 and the number of positions, then so many index steps (lag time values) are generated in an equidistant manner. If nothing, or 0 is given, then values 1…*N*/4 are used.

Each *tau* value results in *M *=* M*(*tau*) pairs, where the step ***r***[*i* + *tau*]−***r***[*i*] is calculated. *M* also depends on whether the set was generated using overlapping intervals (if *overlap *=* True* is set).

If an array of time values (in seconds) are provided for the position data using the optional *tvector* parameter, the algorithm will check the time step between each data pair used. Calculating the mean value of these time steps and using a relative error, every value outside the *mean*(1 ±* tolerance*) will be ignored (by default *tolerance *= 0.1). This process eliminates jumps in the data caused by computer latency during recording.

The function returns a dictionary containing “tau”, “dtau”, “MSD”, “DMSD” keys. If the time values were not provided, then “tau” holds the index steps between the positions and “dtau” is not used.

#### Creep compliance

Assuming that the generalized Stokes-Einstein relation holds, the creep compliance is linearly proportional to the MSD [6]. The *MSD*_*to*_*J*() function calculates the creep compliance using equation (1).

J = MSD_to_J(msd, t0= 0.1, tend = 150,

T = 25.0, a = 1.0)

The calculation requires a dict structure (*msd*) having the time values under the “tau” key, and the MSD values under the “MSD” key (error values are optional). Further parameters are the temperature *T* of the experiment in Celsius degrees, the radius *a* of the applied tracer particle in micrometers, and optionally the dimensionality of the motion *D* (denoted as *N*_*D*_ in equation (1)), which is set to *D *= 2 by default.

Calculating the frequency dependent shear modulus from *J*(*t*) with the numerical method proposed by Evans et al. requires extrapolated values to the zero time point and to infinite time values. These values are estimated here, allowing the user to override them before being used to calculate the complex shear modulus *G*^∗^(*ω*). The zero time value *J*_0_ =* J*(*t *= 0) is extrapolated from a linear fit in the *t *<* t*0 region, and the end extrapolation is obtained from a linear fit to the *tend *<* t* part. The slope of the extrapolated end part is 1/*η*, where *η* is the steady state viscosity [31].

The function returns a new dict containing: “J” (in 1/*Pa*), “tau”, “eta”, “J0”, “const”, “dJ”, and the fit parameters as “a0”, “b0” for the first part and “a1”, “b1” for the end part, where the linear equation *J *=* a*_*i*_*t* + *b*_*i*_(*i *= 0,1) holds.

#### Calculating the frequency dependent complex shear modulus

While the connection presented by equation (2) is simple, there is a major problem with determining $\tilde{J}(\omega )$ numerically. It is well known in numerical analysis that applying a numerical Fourier transform increases the experimental noise enormously [32]. In microrheology, there are four commonly applied methods to solve this problem. The first two address the noise of the Fourier transform directly by averaging or by fitting, the second two were suggested in the last decade to improve the transform itself [6,9,31-33].

In a homogeneous system, where multiple particles can be tracked, converting their MSD to creep compliance and then *G*^∗^(*ω*) using a discrete Fourier transform, allows one to average the converted values and decrease the noise this way [32,33]. For cases when the creep compliance can be modeled using an analytical form, the Fourier transform of the fitted analytical function may be calculated and used to estimate of *G*^∗^(*ω*) [9].

As we have discussed above, samples of biological origin are often not homogeneous and their MSD does not follow a well-described analytical function. However, model calculations suggest, in agreement with experiments, that biopolymers and many polymer gels show power law behavior at various time ranges [34-36]. The third and fourth conversion approaches have been suggested for such systems in the microrheology literature. One uses a power law approximation of the MSD or the creep-compliance [6] (we shall call the Mason method), and the other calculates a linear interpolation between the data points and applying a discrete Fourier transform on it [31] (we shall cite as the Evans method).

Because these methods have their strength and weakness, we summarize them and their implementation below. Recently we have shown that the accuracy of the Evans method can be greatly improved by using local interpolation of the data in a splinelike manner without forcing a single function to be fitted to the whole data set. This improvement is also included in the Rheology framework and will be discussed below.

### The Mason method

This is a fast conversion method based on the Fourier transformation of a power function, which has been used in various works in the last decade [6-8,37-39]. Briefly, let us consider a generalized diffusion process, where the MSD is following a power law: < Δ*r*(*t*)^2^ > = 2*N*_*D*_*Dt*^*α*^[40], where *D* is the generalized diffusion coefficient, and 0 <* α *≤ 2. In the case of *α *= 1 we can talk about regular Brownian diffusion, *α *< 1 describes subdiffusion and *α *> 1 superdiffusion, indicating active forces participating in the process. Using the generalized Stokes-Einstein equation (1) we consider Brownian and subdiffusion processes, thus *α *≤ 1 [5,40]. In this case the creep compliance will also be described with a power function as:

(3) $$J(t)=J_{0}t^{\alpha }$$

**Figure 3:**
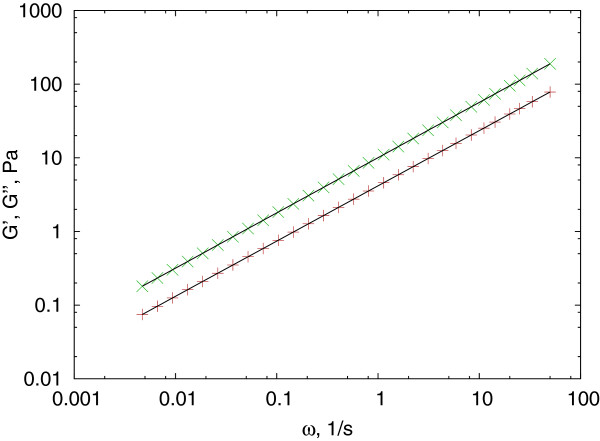
**Testing the Mason method.** Complex shear modulus (storage modulus (red + ) and loss modulus (green ×)) calculated from a power law creep compliance in the form of *J*(*τ*) = 0.1*τ*^0.75^ and converted using the *J*_*to*_*G*_*Mason*() function and theoretically (solid line) using equation (4).

The complex shear modulus can be directly calculated using a gamma function (Figure 3), in the form of:

(4) $$G^{*}(\omega )=e^{\frac{i\Pi \alpha }{2}}\frac{\omega^{\alpha }}{J_{0}\Gamma (1+\alpha )}=\frac{e^{\frac{i\Pi \alpha }{2}}}{J(t=1/\omega )\Gamma (1+\alpha )}.$$

Usually this equation is presented containing the MSD [41], but the key feature, the symmetry between the time dependent creep-compliance and the frequency dependent complex shear modulus, is more apparent in this form. The method generalizes this symmetry between *ω *↔* t *:* ω *= 1/*t*, and assumes it holds for the whole measured time range [6], even when the exponent *α* of the power law varies with time.

However, this is a highly specific case, and the symmetry does not hold for most of the functions [41]. To improve the fit quality, a slightly more complicated version of this formula has been proposed by Dasgupta et al. in [38], based on empirical corrections.

The *J*_*to*_*G*_*Mason*() function implements both methods based on references [6,38] and the *leastsq*() function of scipy, which is a modified Levenberg-Marquardt minimization algorithm.

G = J_to_G_Mason(J,N=30, advanced=True,logstep=True)

The algorithm takes a creep-compliance dict (*J*), and fits a power function locally in the form of equation (3) to estimate *α* and calculates the complex shear modulus at *ω *= 1/*t *using equation (4). The algorithm uses a Gaussian function to weight the neighbors in the local fit as it is described by Mason et al. [6]. *N* defines the desired number of resulted data points, which are created by equidistant sampling in the linear or logarithmic space between 1/*t*_*max*_…1/*t*_*min*_. The logarithmic sampling is activated by the *logstep* parameter, which is set by default. The resulted dict contains ’omega’ and ’f’ for the circular frequency and frequency respectively, and a ’G’ array storing the corresponding complex shear moduli.

There are several further parameters to control the process, from which setting the *advanced* parameter forces the use of the method proposed by Dasgupta et al. instead of the original Mason method, and the *verbose* switch provides graphical feedback on how the local fitting proceeds. A test example is presented in Figure 3 using a creep compliance in the form of equation (3) and converted both numerically and analytically. Because this method is accurate for power law creep compliances, the conversion matches within machine precision.

### The Evans method

The fourth method we again discuss in detail. It is based on the work published by Evans et al. [31]. This method considers a linear interpolation between the *N* data points and provides a conversion in the form of equation (5) [41].

(5) $$G^{*}(\omega )=\frac{i\omega }{i\omega J_{0}+\overset{N}{\underset{k=0}{\sum }}(A_{k+1}-A_{k})e^{-i\omega t_{k}}},$$

where *A*_*i*_s are defined by:

(6) $$A_{k}=\frac{J_{k}-J_{k-1}}{t_{k}-t_{k-1}},\text{where}0<k\leq N,A_{0}=0,A_{N+1}=\frac{1}{\eta }.$$

*J*_0_ and *η* are already estimated using the linear fits in the *MSD*_*to*_*J*() function. Using equation (5) is straightforward, and allow for the calculation of *G*^∗^(*ω*) at any *ω *values. The natural selection of a suitable frequency range would be from 0 to $N\frac{\Pi }{T}$ (the unit is 1/*s*) in *N*/2 steps as it is common for the discrete Fourier transform of equally sampled data [32]. The corresponding function is:

G = J_to_G(J)

**Figure 4:**
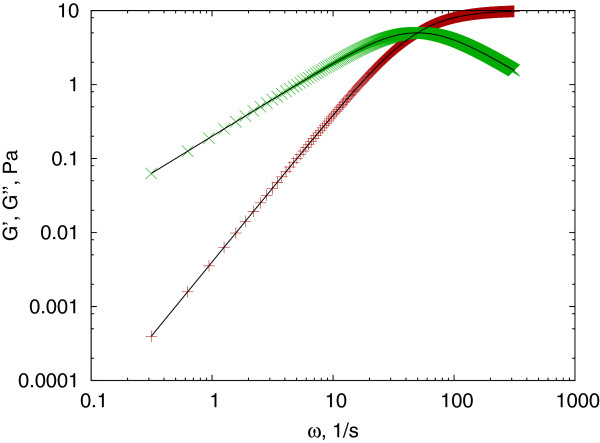
**Maxwell fluid, testing the Evans method.** The complex shear modulus of a Maxwell fluid with Young modulus of *E *= 10*Pa *and viscosity *η *= 0.2*Pas*, calculated using *J*_*to*_*G*() (symbols) and analytically (solid line). The storage modulus (red + ) and loss modulus (green ×) data calculated numerically fits well to the theoretical values presented by the solid black lines.

The algorithm generates a linear array of frequencies, but the number of points is limited to be maximum 1000, usually more than sufficient (MSD and creep compliance data arrays may hold several thousand points). The result is a similar dictionary as it was for the Mason method, and can be tested using a Maxwell-fluid, which has a linear creep compliance in the form of *J*(*t*) = 1/*E* + *t*/*η *(Figure 4).

There are various details worth mentioning about this method, which may affect the accuracy of the result in general cases. It is clear in equation (5), that the method is sensitive to the *A*_*k* + 1_−*A*_*k *_terms, which, in extreme cases, may be either very small for a nearly linear part of *J*(*t*) or very high for sudden jumps in the experimental data. In order to reduce round-off errors, one may eliminate the close to zero values, (when |*A*_*k* + 1_−*A*_*k*_| <* ε*) by activating the *filter* parameter.

**Figure 5:**
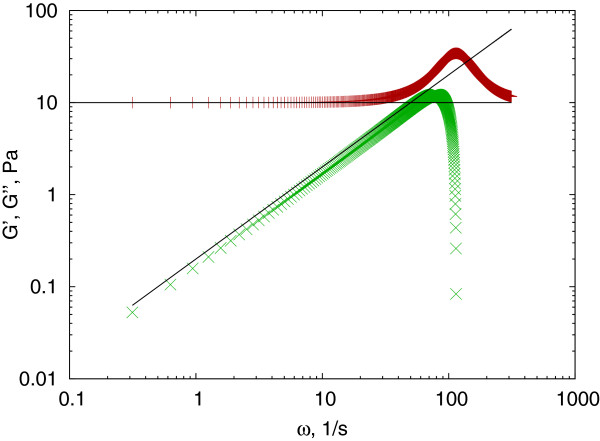
**Kelvin-Voigt solid.** Storage modulus (red + ) and loss modulus (green ×) calculated for a simulated Kelvin-Voigt solid using the *J*_*to*_*G*() function. The numerical results deviate significantly from the theoretical values represented by the solid lines.

This numerical conversion method has two basic problems. First, from equation (5) it is apparent that the complex shear modulus is directly related to the numerical Fourier transform of the *A*_*k* + 1_−*A*_*k *_set, and thus very sensitive to the noise of these data. Second, the limited bandwidth causes the *A*_*k *_values to be a poor representation of the local derivative of the creep compliance, resulting in a strong deviation (usually an unphysical cut-off) of the calculated shear modulus. This latter problem is well represented on a test example of a Kelvin-Voigt solid characterized by a Young’s modulus of *E *= 10*Pa* and viscosity *η *= 0.2*Pas*(Figure 5).

The conversion can be improved by increasing the bandwidth of the data, or decreasing the frequency range where *G*^∗^(*ω*) is calculated (using the *omax* parameter). A third alternative is using model interpolations to increase the bandwidth numerically. Because most experimental data cannot be fitted with a single analytical function for all *τ* values, we have developed a method to fit the MSD at consecutive intervals in a splinelike manner [41].

### Adaptive splinelike fitting

Biopolymer samples commonly show monotonic MSD, frequently described by power laws at various time segments. This makes the choice of the power function a good candidate for the local fitting. To count for deviations from power laws at short time values, the fitting system also allows the use of a Kelvin-Voigt solid as an alternative model for short time scales, in the case of more elastic samples. Including a Maxwell fluid as an option was not necessary, since the Evans method provides perfect fits for linear MSDs (see Figure 4).

Because this algorithm has not been published previously, we describe it here in detail. The procedure is designed to run automatically being controlled only by selecting the start interval of the data and a scaler, which defines how fast the fitting range should increase with time. The key steps are: 

1. define the data range using indices *i*_0_ = 0 and *i*_1_ =* i*(*t*_0_), containing at least 4 data points;

2. fit function from *i*_0_…*i*_1_, calculate the squared error of each point and estimate the average error;

3. find the last point around *i*_1_ where $\left(\chi^{2}(i_{2})<\chi_{\text{mean}}^{2}\right)$ and redefine *i*_1_ as this point *i*_1_ =* i*_2_(stretching or shrinking the fitting range to a reasonable maximum)

4. recalculate the fit and errors, store as the current segment

5. if we reached the end (or close to the end), finish the cycle, return with the results

6. otherwise, define the new fitting range, using the *scaler* parameter (by default use *i*_0_…*scaler *×* i*_0_)

7. return to 2 (see Additional file 1)

The exact procedure calculating the new range in each step may vary depending on the *mode* an optional keyword parameter, allowing for some control for functions which show fast changes or slow changes with noise. The default method (*mode*=“scaler”) described above works well for most MSDs with some noise but monotonic trends. (More details are available in the help of the library and the *config.example* text file in the Examples folder of the package).

The corresponding function call is:

fits = MSD_power_fit(msd, t0=0.2,scaler=10.0).

Completing the above algorithm, in the next step the program checks where the fits would cross each-other in the neighboring ranges, and readjusts them to the crossing point, if it lies within the union of the two ranges. This step helps to maintain a smoother approximation of the experimental data. Turning verbosity on using *verbose=True*, one may see details about how the algorithm operates.

### Dynamic and static interpolation

The return value (*fits*) is a list of dicts, each containing the fitting parameters of one segment. This list can be directly used to calculate the interpolation of the original data using:

msd2 = MSD_interpolate(msd, fits, smoothing

= True, Nsmoothing=10, insert=10, factor=3)

The interpolation and insertion of new points before the first data point will increase the bandwidth of the original data. The *factor* parameter controls the oversampling of the original data (here it is set to 3× oversampling). Inserting new data points between 0 and the first time *τ*_0_ is specified by *insert *= 10.

Estimating how many new points are required during this resampling procedure is a difficult question in general. The above example uses a static approach, simply inserting *factor* points between every two original data points, which results in a very large equidistant data set. Alternatively we provide a dynamic method, which is controlled by an error parameter.

Investigating the form of equations (2) and (6), one can see that the accuracy of the Evans method is strongly related to how accurately equation (6) approximates the local derivative of *J*(*t*). Knowing the analytical form of the interpolating functions, this error can be approximated using the function (*f *) and its second derivative (*f *”) [32,41]. Based on this approximation and specifying a local error *ε*, the minimal step size at any time point *h*(*t*) can be estimated as:

(7) $$h=\varepsilon \sqrt{\frac{f_{k}(t_{k})}{{f_{k}}^{\prime \prime }(t_{k})}}.$$

Using *h* as a minimal step size for each fitted segment, the program can dynamically interpolate the data and increase the number of points only where the creep compliance changes faster. This results in a non-equally sampled data set, which (after calculating the creep compliance) can be well handled by both the *MSD*_*to*_*J*() function and subsequently the numerical conversion method *J*_*to*_*G*(), resulting in an improved complex shear modulus. To eliminate further bandwidth problems, the maximal desired circular frequency *ω*_*max*_ can be forced to $\frac{N\Pi }{T}$ using the *omax* parameter.

**Figure 6:**
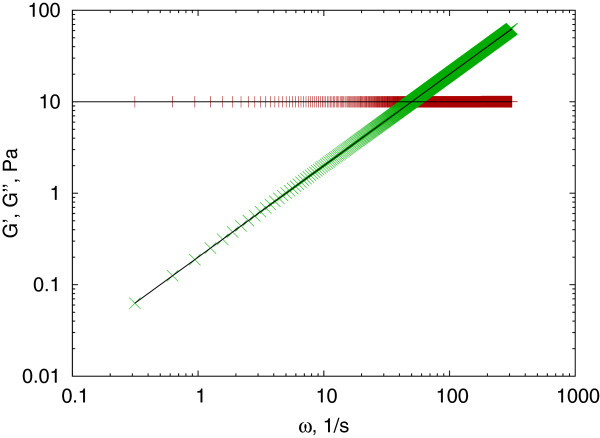
**Dynamic resampling.** Dynamic resampling can correct the errors of the Evans method resulting in an improved fit. The storage modulus (red + ) and loss modulus (green ×) data calculated numerically shows a good fit to the theoretically predicted values (black lines).

The consequence of this fitting and interpolation procedure is an increased bandwidth and decreased noise in the interpolated data set. The resulting accuracy is some orders of magnitude larger than the specified *ε *but strongly related to it. Therefore the user has to estimate the suitable *ε *for the given problem. For example, inserting 10 new points between 0−*t*_0_ and requesting an accuracy of *ε *= 5×10^−4^, the algorithm has corrected the errors of Figure 5 to about 1% relative error on average (Figure 6). The Mason method produces about 100% relative error for the loss modulus of the same conversion, originating from the failure of its power law assumption, and can not be improved by interpolation [41].

The advantage of employing non-equally sampled data as the result of the dynamic interpolation is clear if we compare the required smallest time step produced by this method. The results indicate *h*_*min*_ ≈ 2 × 10^−6^* s*, which would increase the number of data points about 5000× in the case of equidistant sampling. In comparison, the increment of the number of data points is only about 20%.

For data sets following various trends in the different time segments (thus the fitting procedure identifies multiple fitted regions), the transition between the regions can be smoothened using a linear interpolation between the two smoothing functions. The range of this smoothing is defined by the *NSmooth* parameter. The range is identified in the original data, but then applied to the refined data set. The result is an MSD, where sudden jumps are reduced, minimizing the presence of oscillatory artifacts in the resulted complex shear modulus *G*^∗^(*ω*).

## Conclusions

In this paper we have presented a free software solution for analyzing particle tracking data for microrheology. Our software library implements the time average calculation of mean square displacement with control over the time shift in the data, and conversion methods to calculate the creep compliance and the complex shear modulus. Beyond the two most common methods mentioned in the literature, we have developed a dynamic local fitting procedure, which allows spline-like fitting of the MSD and improved conversion accuracy to about 1% from about 100% for a Kelvin-Voigt model test.

## Availability and requirements

Lists the following: 

• **Project name:** Rheology for Python

• **Project home page:**http://launchpad.net/microrheologypy/

• **Operating systems:** Platform independent (Linux, Windows and Max OSX tested)

• **Programing language:** Python 2.7

• **Other requirements:** Numpy 1.5, Scipy 0.1, Matplotlib 1.0

• **License:** LGPL v3

• **Any restrictions to use by non-academics:** see license

## Abbreviations

MSD: Mean square displacement.

## Competing interests

The Authors declare that they have not competing interests.

## Authors’ contributions

TM and TH have developed the Python software together discussing and testing the various features, and both contributed to writing this manuscript. Both authors read and approved the final manuscript.

## Supplementary Material

Additional file 1 **rheology.zip - compressed ZIP archive containing the Rheology Python package.** The archive contains several files. The source code in the Rheology subfolder, a setup.py for installation, README.txt and License.txt files and the Example subfolder. Installation (as usual in python):
python setup.py build; python setup.py install
The Example subfolder contains two Python scripts. The Function-test.py can be used to run test calculations and see the example plots presenting that all functions work properly. The figures presented in this paper were also generated by this script.
The ProcessRheology.py is a batch processing script, controlled by commands and parameters provided in a config.txt plain text file. An example of this file is also included here, containing detailed description of every parameter. This scripts is a fully functioning microrheology data evaluation toolkit, utilizing the functions of the Rheology package. Click here for file

## References

[B1] Dangaria JHH Butler PJJ Macrorheology and adaptive microrheology of endothelial cells subjected to fluid shear stress Am J Physiol Cell Physiol 2007 293 C1568 C1575 10.1152/ajpcell.00193.2007 17670893 PMC3251213

[B2] Bausch AR Moller W Sackmann E Measurement of local viscoelasticity and forces in living cells by magnetic tweezers Biophys J 1999 76 573 579 10.1016/S0006-3495(99)77225-5 9876170 PMC1302547

[B3] Waigh TA Microrheology of complex fluids Rep Prog Phys 2005 68 3 685 10.1088/0034-4885/68/3/R04 27245584

[B4] Wirtz D Particle-tracking microrheology of living cells: principles and applications Annu Rev Biophys 2009 38 301 326 10.1146/annurev.biophys.050708.133724 19416071

[B5] Mason TG Weitz DA Optical measurements of frequency-dependent linear viscoelastic moduli of complex fluids Phys Rev Lett 1995 74 7 1250 1253 10.1103/PhysRevLett.74.1250 10058972

[B6] Mason TG Ganesan K van Zanten JH Wirtz D Kuo SC Particle tracking microrheology of complex fluids Phys Rev Lett 1997 79 3282 3285 10.1103/PhysRevLett.79.3282

[B7] Mason TG Estimating the viscoelastic moduli of complex fluids using the generalized Stokes-E, instein equation Rheologica Acta 2000 39 4 371 378 10.1007/s003970000094

[B8] Squires TM Mason TG Fluid mechanics of microrheology Annu Rev Fluid Mech 2010 42 413 438 10.1146/annurev-fluid-121108-145608

[B9] Goodwin JW Hughes RW Rheology for Chemists. An Introduction 2008 Cambridge: RSC Publishing

[B10] Mezger TG The Rheology Handbook. 3rd revised edition. Vincentz Network GmbH & Co. KG , Plathnerstr. 4c 2011 Hannover, Germany: European Coatings Tech Files, Vincentz Network

[B11] Crocker J Weeks E Microrheology tools for IDL [ http://www.physics.emory.edu/weeks/idl/rheo.html]

[B12] Kilfoil M Matlab algorithms from the Kilfoil lab [ http://people.umass.edu/kilfoil/downloads.html]

[B13] Tassieri M Compliance to complex moduli, version 2 2011 [ https://sites.google.com/site/manliotassieri/labview-codes]

[B14] Jones E Oliphant T Peterson P SciPy: Open source scientific tools for Python 2001 [ http://www.scipy.org/]

[B15] Hunter JD Matplotlib: A 2D graphics environment Comput Sci Eng 2007 9 3 90 95 [ http://matplotlib.sourceforge.net/]

[B16] Crocker JC Grier DG Methods of digital video microscopy for colloidal studies J Colloid Interface Sci 1996 179 298 310 [ http://www.physics.emory.edu/weeks/idl/] 10.1006/jcis.1996.0217

[B17] Brangwynne CP Koenderink GH Barry E Dogic Z MacKintosh FC Weitz DA Bending dynamics of fluctuating biopolymers probed by automated high-resolution filament tracking Biophys J 2007 93 346 359 10.1529/biophysj.106.096966 17416612 PMC1914425

[B18] Gosse C Croquette V Magnetic tweezers: micromanipulation and force measurement at the molecular level Biophys J 2002 82 6 3314 3329 10.1016/S0006-3495(02)75672-5 12023254 PMC1302119

[B19] Rogers SS Waigh TA Lu JR Intracellular microrheology of motile amoeba proteus Biophys J 2008 94 8 3313 3322 10.1529/biophysj.107.123851 18192370 PMC2275677

[B20] Carter BC Shubeita GT Gross SP Tracking single particles: a user-friendly quantitative evaluation Phys Biol 2005 2 60 10.1088/1478-3967/2/1/008 16204858

[B21] Savin T Doyle PS Statistical and sampling issues when using multiple particle tracking Phys Rev E 2007 76 2 021501 10.1103/PhysRevE.76.021501 17930038

[B22] Savin T Doyle PS Static and dynamic errors in particle tracking microrheology Biophys J 2005 88 623 638 10.1529/biophysj.104.042457 15533928 PMC1305040

[B23] Savin T Spicer PT Doyle PS A rational approach to noise discrimination in video microscopy particle tracking App Phys Lett 2008 93 2 024102 10.1063/1.2957464

[B24] Smith R Spaulding G User-friendly, freeware image segmentation and particle tracking [ http://titan.iwu.edu/gspaldin/rytrack.html]

[B25] Blair D Dufresne E The Matlab particle tracking code respository 2005 2008 [ http://physics.georgetown.edu/matlab/]

[B26] Milne G Particle tracking 2006 [ http://zone.ni.com/devzone/cda/epd/p/id/948]

[B27] Caswell TA Particle identification and tracking [ http://jfi.uchicago.edu/tcaswell/track_doc/]

[B28] Haraszti T ImageP: image processing add-ons to Python and numpy 2012 2009 [ https://launchpad.net/imagep]

[B29] Flyvbjerg H Petersen HG Error estimates on averages of correlated data J Chem Phys 1989 91 461 466 10.1063/1.457480

[B30] Saxton M Single-particle tracking: the distribution of diffusion coefficients Biophys J 1997 72 4 1744 1753 10.1016/S0006-3495(97)78820-9 9083678 PMC1184368

[B31] Evans RML Tassieri M Auhl D Waigh TA Direct conversion of rheological compliance measurements into storage and loss moduli Phys Rev E 2009 80 012501 10.1103/PhysRevE.80.012501 19658751

[B32] Press WH Teukolsky SA Vetterling WT FB P Numerical recipes in C++ 2002 Cambridge: Cambridge University Press

[B33] Addas KM Schmidt CF Tang JX Microrheology of solutions of semiflexible biopolymer filaments using laser tweezers interferometry Phys Rev E 2004 70 2 021503 10.1103/PhysRevE.70.021503 15447492

[B34] Morse DC Viscoelasticity of concentrated isotropic solutions of semiflexible polymers. 2. Linear Response Macromolecules 1998 31 20 7044 7067 10.1021/ma980304u

[B35] Morse DC Viscoelasticity of concentrated isotropic solutions of semiflexible polymers. 1. model and stress tensor Macromolecules 1998 31 20 7030 3043 10.1021/ma9803032

[B36] Gittes F Schnurr B Olmsted PD MacKintosh FC Schmidt CF Microscopic viscoelasticity: shear moduli of soft materials determined from thermal fluctuations Phys Rev Lett 1997 79 17 3286 3289 10.1103/PhysRevLett.79.3286

[B37] Crocker JC Valentine MT Weeks ER Gisler T Kaplan PD Yodh AG Weitz DA Two-point microrheology of inhomogeneous soft materials Phys Rev Lett 2000 85 4 888 891 10.1103/PhysRevLett.85.888 10991424

[B38] Dasgupta BR Tee SY Crocker JC Frisken BJ Weitz DA Microrheology of polyethylene oxide using diffusing wave spectroscopy and single scattering Phys Rev E 2002 65 5 051505 10.1103/PhysRevE.65.051505 12059562

[B39] Cheong F Duarte S Lee SH Grier D Holographic microrheology of polysaccharides from streptococcus mutans biofilms Rheologica Acta 2009 48 109 115 10.1007/s00397-008-0320-1

[B40] Tejedor V Benichou O Voituriez R Jungmann R Simmel F Selhuber-Unkel C Oddershede LB Metzler R Quantitative analysis of single particle trajectories: mean maximal excursion method Biophys J 2010 98 7 1364 1372 10.1016/j.bpj.2009.12.4282 20371337 PMC2849086

[B41] Maier T Boehm H Haraszti T Splinelike interpolation in particle tracking microrheology Phys Rev E 2012 86 011501 10.1103/PhysRevE.86.011501 23005419

